# Evidence for the Adverse Effect of Starvation on Bone Quality: A Review of the Literature

**DOI:** 10.1155/2015/628740

**Published:** 2015-02-24

**Authors:** Janina Kueper, Shaul Beyth, Meir Liebergall, Leon Kaplan, Josh E. Schroeder

**Affiliations:** ^1^Charité University of Medicine, Charitéplatz 1, 10117 Berlin, Germany; ^2^Department of Orthopedic Surgery, Spine Surgery, Hadassah Medical Center, Kiryat Hadassah, P.O. Box 12000, 91120 Jerusalem, Israel

## Abstract

Malnutrition and starvation's possible adverse impacts on bone health and bone quality first came into the spotlight after the horrors of the Holocaust and the ghettos of World War II. Famine and food restrictions led to a mean caloric intake of 200–800 calories a day in the ghettos and concentration camps, resulting in catabolysis and starvation of the inhabitants and prisoners. Severely increased risks of fracture, poor bone mineral density, and decreased cortical strength were noted in several case series and descriptive reports addressing the medical issues of these individuals. A severe effect of severely diminished food intake and frequently concomitant calcium- and Vitamin D deficiencies was subsequently proven in both animal models and the most common cause of starvation in developed countries is anorexia nervosa. This review attempts to summarize the literature available on the impact of the metabolic response to Starvation on overall bone health and bone quality.

## 1. Introduction

Starvation describes the most severe form of malnutrition, where a severe deficiency in energy intake evokes a metabolic response focused on the subsistence of the vital organs to allow for the survival of the affected individual. Nearly 805 million people are estimated to suffer from malnutrition. 25% of children experience stunted growth due to malnutrition, whilst approximately 45% of deaths in children under five can be correlated with starvation [1, 2].

### 1.1. Causes of Starvation

Starvation may be caused either by an insufficient caloric intake or an inability to properly digest food. Environmental circumstances such as draughts or other natural catastrophes affecting the agriculture, poverty, or forceful withholding in certain geopolitical circumstances such as war or political prison camps may contribute to the unavailability of food. This occurs most commonly in less developed countries. In more developed countries, the primary causes of starvation are medical. Diseases such as anorexia nervosa or depression which lead to a self-induced lack of food intake are not uncommon causes of starvation if the diseases are not diagnosed and treated correctly.

### 1.2. Metabolic Response to Starvation

The initial metabolic response to starvation does not differ physiologically from the postabsorptive phase in between meals which may usually be observed in a well-nourished human being [3, 4]. The body relies on the dietary glucose supplied by food intake initially, switching to fatty acids once all dietary glucose has been absorbed and utilized. Although most of the body can subsist on the breakdown of fatty acids, the skin, kidney medulla, erythrocytes, and the brain amongst others require glucose for their metabolism [5]. To maintain a steady concentration of glucose in the blood stream, excess dietary glucose previously stored in the liver as glycogen is reduced back to glucose [6]. Once this glycogen storage of approximately 120 grams is used up, the body must revert to gluconeogenesis. This process utilizes mostly glutamine and alanine with glycerol to produce glucose in the liver, kidney, and intestine [7, 8]. In parallel, the production of ketones such as 3-hydroxybutyrate and acetoacetate, substrates which are able to supply the brain as they are able to cross the blood-brain barrier, is initiated in the liver [9, 10]. With no additional food intake, the body slowly adapts, relying primarily on triglycerides deposited in adipose tissue and amino acids stored in smooth-, cardiac-, and skeletal muscle as sources for its metabolism. Starvation ensues when protein remains the only source of energy for the body. The amount of glucose usually utilized by the body is reduced to a minimum, with the metabolic rate of the cells being decreased significantly to allow for the subsistence of the organism as a whole [11–13]. Individuals who suffer from chronic starvation adapt, displaying similar basal metabolic rates as healthy individuals when adjusted for fat free mass since the visceral organs with the highest metabolic rates such as the brain and the kidneys remain relatively unaffected [14, 15]. Both the reduction of the basal metabolic rate as well as the commonly present vitamin and nutrient deficiencies have been hypothesized to contribute to stunted growth, bad bone quality, and an earlier onset of osteoporosis in later life.

## 2. Evidence for the Adverse Effect of Starvation on Bone Quality

Starvation may occur for either limited periods of time followed by a return to a regular food intake or subsist over extended periods of time, thereby leading to a chronic adaptation to the low caloric intake or absorption.

### 2.1. Animal Studies

Starvation induced changes of the bone have been described and experimented with in various animal models.

The most naturally occurring physiologic cause of starvation which can be observed in nature occurs during the hibernation of black, brown, and polar bears. Osteoblastic activity levels have been reported to decrease tremendously during hibernation, caused most likely by both immobility and starvation [16]. Nonetheless, the bone area, bone mineral density, and cortical strength have been shown to show little change when compared to the period of time the bear does not spend in hibernation, returning to baseline after a short period of remobilization [17–20]. Various additional mechanisms including maintenance of osteoblastic bone formation, increased parathyroid hormone levels and differential expression of genes responsible for osteoclast formation and differentiation such as Ostf1, Rab9a, and c-Fos have been discussed as causes of this phenomenon [21–23]. A reduction of the baseline metabolic rate by 25% and the upregulation of the expression of anabolic genes of the skeletal muscle- and cartilage metabolism have been hypothesized to be contributors as well [23, 24]. Additional research with greater differentiation between the effect of the starvation and the effect of the immobility on the bone metabolism and its physiologic countereffect may allow for greater insight. Similar nature based studies have been performed with moose in Norway. The projects were initiated to determine the cause of a high incidence of fractures and osteoporosis in the moose population of Norway. Moose that had generalized Osteoporosis with a decreased bone mass, bone mineral density, and cortical strength were found to have the lowest carcass weights of their population most likely associated with starvation caused through overcrowding and an increased competition for feed [25, 26]. As opposed to bears, Moose show no adaptation of bone metabolism to starvation and exhibit bone opacity reduced almost in half in animals subject to food deprivation.

Prospective animal studies most commonly performed in a rat model have shed great light on the effect of energy restriction and starvation on fetal bone development in utero, associated hormones and consequences for the adult animal. Hermanussen et al. [27] demonstrated that stunted growth of long bones in both intact and GH-deficient rats induced by starvation was not repairable through a reinitiating of feeding as the growth spurts responsible for growth simply ceased during starvation and did not increase once feeding was commenced. Banu et al. [28, 29] were able to demonstrate a significant loss of endocortical bone as well as cancellous bone area and cancellous bone mineral content in rats with food restrictions in addition to concomitant decreases in tibial muscle mass. Swift et al. [30] reported that food restrictions in their rat model led to the greatest decrease in bone mineral density in the cancellous bone of the tibia in comparison to restrictions of energy or calcium intake. The total body mineral content was found to be reduced by 13% whilst the total volumetric bone mineral density at the proximal tibia metaphysic were found to be reduced by 8% compared with rats who received ad libitum access to their food and exercise. Talbott et al. [31] examined the effect of food and calcium intake on younger (3 months) and older (10 months) rats and found that both restrictions generally led to a higher rate of bone turnover measured by urinary [3H]TC excretion. Solely older animals however were found to have decreased bone mineral density resulting from the food restriction. Overall, animals with a calcium and energy controlled diet lost approximately 0.5% bone mineral density whilst control animals experienced an increased bone mineral density by more than three percent. Engelbregt et al. examined the effect of pre- and postnatal malnutrition on bone composition [32]. They found that both pre- and postnatally deprived rats demonstrated a decreased total body mineral content by a mean 0.4 and 0.9 grams, respectively, when compared to the mean total body mineral content of control animals-2.4 grams. Romano et al. [33] reported on adult bone quality of rats exposed to malnutrition in utero through the creation of an artificial uteroplacental insufficiency after calcium supplementation treatment. They found that, regardless of the supplementation, rats that experienced the consequences of the insufficiency in utero demonstrated a body weight decreased by up to 14%, decreased trabecular and cortical bone mineral density, decreased femur length, and decreased stress strain index by up to 9% compared to control animals.

Overall, the observations in wild moose and laboratory rats with regards to the metabolic bone response to starvation mimic human physiology more than the hibernating bear. A greater level of understanding of bear's ability to maintain their bone mineral density despite 5–8 months of immobility and starvation may inspire new studies investigating the effect of starvation on human bone.

### 2.2. Famine: Starvation in Utero

Malnutrition and starvation endured during famine may affect not only children and adults, but also fetuses in utero. The possibility of intrauterine programming of musculoskeletal disease developed in the adult human being was initially proposed by Lucas [34]. It is generally assumed that the earlier in the development of the fetus the maternal malnutrition or starvation takes place, the greater the effect on the bone mass and quality at birth [35]. Due to obvious ethical concerns, few studies have been able to illustrate a direct connection in between maternal malnutrition and starvation and infantile and adult bone health. Despite this, several projects have been able to correlate factors indicative of decreased bone health in the neonate, infant and adult with low maternal Vitamin-D levels and food intake. The osteoblastic invasion of the cartilaginous skeleton during the 5th week of pregnancy may be adversely affected by calcium and Vitamin D deficiencies of the mother, leading to osteopenia in the adult later in life [36]. Namgung et al. further described the associations between the low maternal Vitamin D-status and decreased bone mineral content as well as increased bone resorption in the infant [37–39]. They found higher serum osteocalcin and 1,25(OH)2D, higher serum cross-linked carboxy-terminal telopeptide of type I collagen and lower serum total calcium in children born in winter when compared to children born in summer. Additionally, children born in winter demonstrated a total body mineral content decreased by 8% when compared to children born in summer. Cooper et al. found that low birth- and infant weight as well as diminished growth rates correlate with both a decreased bone mineral content and an increased risk of hip fractures in later life [40–42]. Bone mineral content of the spine was found to correlate with infant weight at one year in both women and men. Low childhood growth rates, particularly with regards to height, were closely correlated with an increased hazard ratio for hip fracture. Furthermore, low birth and infant weight have been associated with decreased Growth Hormone- and Cortisol levels in the adult-factors usually protective of bone health through the inhibition of bone demineralization [43–45]. Neonatal bone mass has been determined to be independently predicted by the maternal food intake at 18 weeks of gestation [46].

### 2.3. Famine: Starvation as an Adult

**Figure 1:**
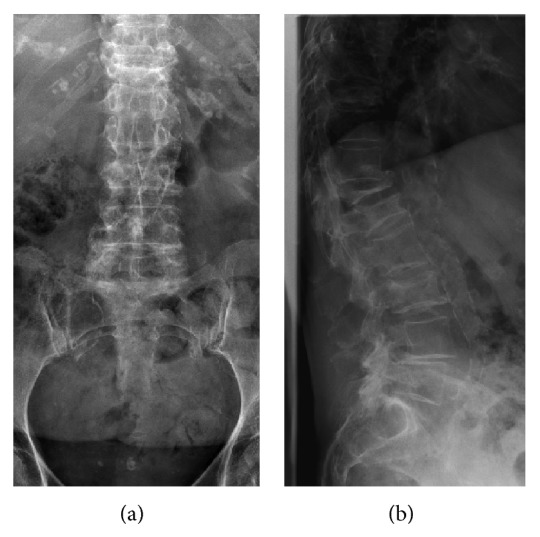
AP and lateral images of the lumbar spine of a 78-year-old female, Holocaust survivor with severe osteoporosis and multiple compression fractures. The latest fracture happened when the patient sneezed. Demonstrating the severe osteoporosis.

The effects of periods of acute starvation in the child or the adult were discussed in several case series examining the potential effect of times of deprivation and malnutrition on the overall bone quality, incidence, and time of onset of osteoporosis, and risk of fracture. Immediate effects on overall bone health were reported by Winnick [47], detailing the observation of Jewish Physicians working in the Warsaw Ghetto in 1941-1942. Fractures in children who survived infancy in the early years of the Ghetto reportedly did not heal, compared to an exceedingly low incidence of pseudoarthrosis of pediatric fractures in healthy young children. The children themselves exhibited severely stunted growth [48]. Autopsies of adult victims of starvation were noted to demonstrate severe demineralization of the cortex and matrix decomposition. One may assume that the conditions and subsequent bone health of prisoners of concentration camps fared no better. Additional case series examining small numbers of survivors of the Holocaust and the Budapest Ghetto reported a higher risk of an earlier onset and more severe form of osteopenia and osteoporosis as well as an increased risk of fractures [49–51]. In the only comparative study by Marcus and Menczel, 73 female Jewish Holocaust survivors aged 60 and above were matched with Jewish females from Europe aged 60 and above uninvolved in the Holocaust. The bone mineral density of both groups was assessed in the lumbar spine and hips. Holocaust survivors were found to have osteoporosis in 54.8% of the cases and osteopenia in 39.7% compared to the control group with a 25.0% incidence of osteoporosis and a 55.0% incidence of osteopenia. The incidence of osteoporosis and osteopenia was found to be especially high in Holocaust survivors aged younger than 17 by the conclusion of World War II [52]. With a daily caloric allowance of 200–800 spanning World War II and potentially several years of the postwar period, the effect of periods of acute starvation lasting several years on bone health were not to be erased by a relative return to nutritional normalcy in any of the case series discussing Holocaust and Ghetto survivors (Figures 1(a) and 1(b)). Similar case series examining bone health of survivors of the Dutch famine. Few studies that do not focus on a very small number of survivors of famine exist; Kin et al. [53] confirmed the results found by these case series in their large series examining 1,826 Chinese women aged 65 years and older. They found that women who had experienced famine at some point in their life exhibited a higher incidence of osteoporosis. The rate of developing osteoporosis in women who had at some point in their lives experienced starvation was increased by 5.3% when compared to women who had not. In addition, they found that women who had experienced starvation had decreased femoral neck bone mineral content and bone mineral density, a lower socioeconomic status and educational level and decreased height.

### 2.4. Anorexia Nervosa

One of the most commonly examined diseases with regards to its effect on bone health is anorexia nervosa, a psychiatric disorder more common in females than in males characterized by a self-induced restriction of caloric intake coupled with a variety of other possible symptoms such as distorted self-perception and compulsive eating rituals [54, 55]. Possible physiologic consequences of anorexia nervosa include classic symptoms of starvation: weight loss, muscle wasting, and amenorrhea. Osteoporosis has been hypothesized to be associated with anorexia nervosa as a possible consequence of the lack of estrogen, calcium and Vitamin D deficiencies, hypercortisolemia, or duration of the illness [56–60]. Bulimia, another eating disorder characterized by binge eating and subsequent vomiting, has not been implicated to have an effect on bone health in comparison with anorexia nervosa [61, 62]. The self-induced starvation of anorexia nervosa has been implicated as an increasing contributor to the development of osteoporosis in the young [63]. Several studies have found a significantly decreased bone mineral density as well as an increased rate of bone resorption and a decreased rate of bone formation [62, 64, 65]. Sokya et al. found increased serum levels of osteocalcin, bone-specific alkaline phosphatase, deoxypyridinoline, and N-telopeptide in adolescent girls diagnosed with anorexia nervosa when compared to healthy control subjects even after a recovery period of one year [65]. In extreme cases of chronic starvation caused by ongoing anorexia nervosa, the decrease in the quality of both the cortical- and the trabecular bone may result in stress fractures, particularly in combination with strenuous exercise frequently performed by affected individuals [66, 67]. Maugars et al. reported on five females who were diagnosed with anorexia nervosa through the discovery of osteoporotic vertebral compression fractures or peripheral insufficiency fractures after a disease duration spanning 7–24 years [67]. Hypercaloric nutritional therapies in combination with calcium- and Vitamin-D-supplementation have been demonstrated to be helpful in restoring bone health. Heer et al. administered hypercaloric diets, high calcium intake (2000 mg/day), and Vitamin D (400 IU/day) to 19 female patients diagnosed with anorexia nervosa. After 11 weeks, they found an increase of the BMI by a mean 2.9 points, an almost doubled amount of the serum bone formation markers bone formation markers procollagen-I carboxy-terminal propeptide and bone specific alkaline phosphatase and a significantly decreased serum concentration of the bone resorption marker C-telopeptide [68]. Oral contraceptive therapy in itself has not been deemed successful in improving bone density in either osteopenic or osteoporotic patients diagnosed with anorexia nervosa [69–71]. However, estrogen replacement therapy has shown promising results if administered in combination with low doses of recombinant human IGF-1 (rhIGF-1). Grinspoon et al. found that female Anorectic patients who received rhIGF-1 showed markedly increased serum markers of bone formation and decreased serum markers of bone resorption 6 days after treatment [72]. In a followup study, they examined the effect of either the administration if rhIGF-1, an oral contraceptive containing ethinyl estradiol and norethindrone, a combination of either treatments or no treatment at all on the bone density of the spine in 60 osteopenic women diagnosed with anorexia nervosa. At 9-month followup, they found that the combination of the contraceptive and the rhIGF-1 lead to the most significant increase in bone density-1.5% higher than in patients receiving no treatment whatsoever [73]. Overall, Hartman et al. [74] examined a series of women who had recovered from anorexia nervosa and found that bone mineral density remained decreased a mean 21 years after recovery.

## 3. Conclusion

Overall, all studies examining the connection between starvation and the bone metabolism in laboratory animal models and humans found evidence of either developmental delays, stunted bone growth, decreased bone mineral density or decreased cortical strength. Given the importance of good bone health to the mobility and function of every human being, public health research investigating the prevention of starvation as well as research focusing on the optimization of therapeutic options for those who have endured periods of famine is in order.

## Core Tip

Malnutrition and Starvation have long been speculated to play a role in the development of osteopenia and osteoporosis. The biochemical and physiologic consequences of prolonged periods of malnutrition and starvation endured in utero, as an infant, child, adolescent, or adult are grave as the body ensures primarily the nourishment of vital visceral organs such as the brain, heart, and kidneys. Starvation has been proven to decrease growth and bone mineral density in animal models. Case series examining the bone health of survivors of the Holocaust or the Ghettos as well as patients diagnosed with anorexia nervosa have demonstrated similar results.
